# Non-syndromic aortic valve myxoma in a teen, cause of angina symptoms

**DOI:** 10.1186/s13019-019-0968-x

**Published:** 2019-07-23

**Authors:** Geoffrey Joseph Changwe, Tao Zhang, Haizhou Zhang, Chengwei Zou

**Affiliations:** Departments of Cardiovascular Surgery and Ultrasound, Shandong Provincial Hospital affiliated to Shandong University, Jingwu No. 324, 250021 Jinan, People’s Republic of China

**Keywords:** Cardiac myxoma, Aortic valve myxoma, Non-syndromic, Carney complex

## Abstract

**Background:**

Cardiac myxoma, a common benign primary tumor of the heart can be categorized into syndromic (Carney Complex) and non-syndromic(isolated). Carney Complex associated myxomas can be found in any region and system (cardiac, cutaneous, osseous, genitalia), and may manifest at a tender age. On the contrary, non-syndromic cardiac myxomas are usually confined to the chambers, and symptoms often present from 5th decade of life. Aortic valve myxoma is a very unusual occurrence, and presentation in a teen is extremely rare.

**Case report:**

We share a case of aortic valve myxoma, uncovered using echocardiography in a 16-year-old male, admitted with complaints of exertional chest pain, dyspnoea and systolic murmur. Patient underwent uneventful surgery for tumor excision, and discharged 6-days post operation.

**Conclusion:**

Given the high risk of developing cardiogenic stroke, infective endocarditis, degenerative effects on aortic valve leaflets and possible sudden death, like many other centers, we advocate for immediate liquidation of aortic myxoma regardless of age and symptoms.

## Background

Aortic valve myxomas are a very rare finding of the non-syndromic primary benign cardiac tumors, and presentation in teen patients is extremely unusual. If not liquidated on time can cause a spectrum of complications [1–4] and sudden death.

## Case presentation

**Fig. 1:**
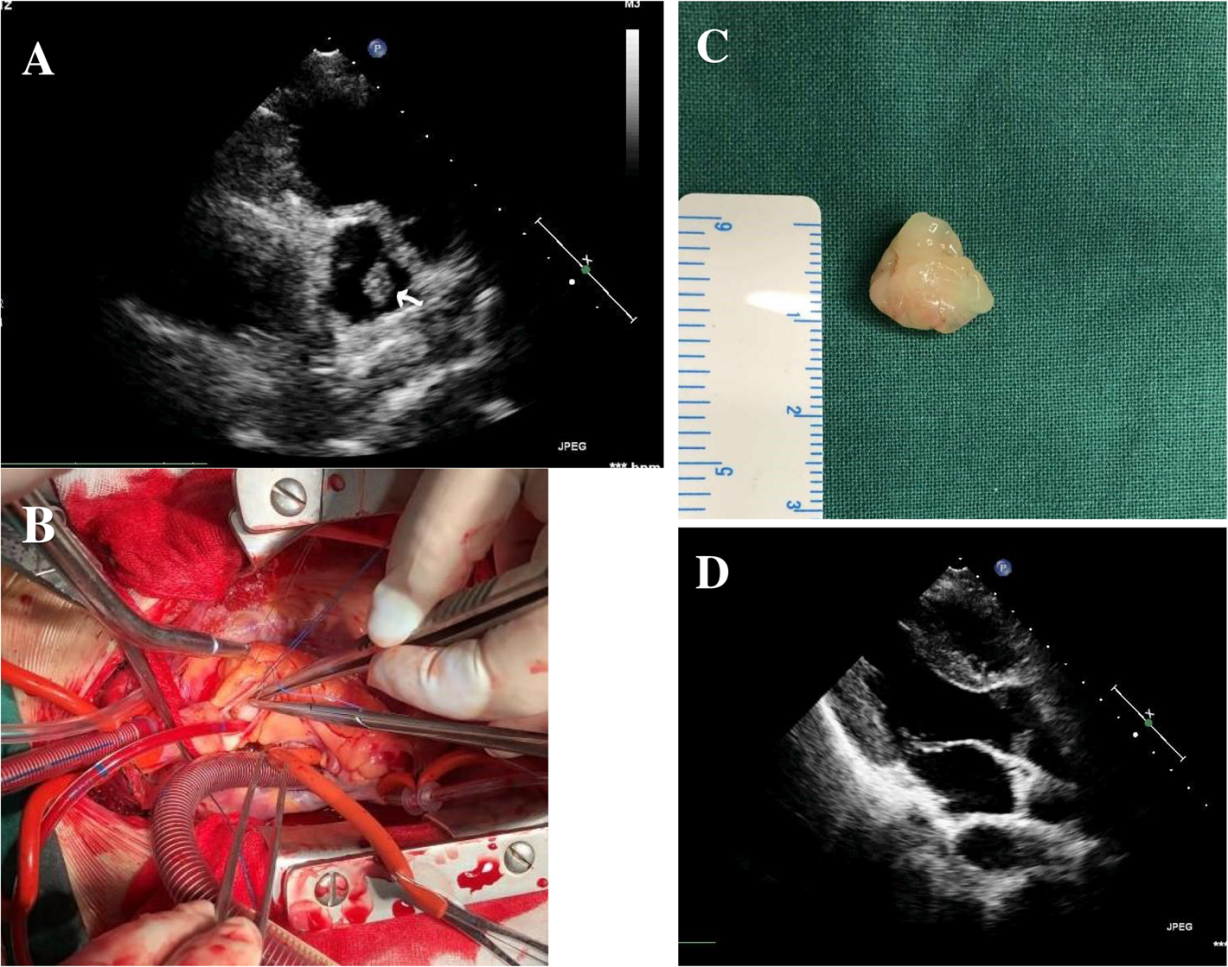
Panels **a** & **d**, pre/post operation echocardiography (white arrow-myxoma), **b**-aortotomy & **c** –myxoma

A 16-year-old male patient presented to our cardiology department with a 4-month history of exertional chest pain and dyspnea. It is alleged that, sometime in March,2018, the patient suffered from a ‘common-cold’, which presented in form of chest pain and high body temperature. Despite having undergone standard treatment and complete liquidation of both fever and chest pain at rest, any physical stress begun eliciting chest pain. On physical examination of the skin and genitalia excluded features associated with Carney Complex [3], while, auscultation revealed a systolic murmur in the 2nd right intercostal pace, which slightly muffled upon supine positioning. Clinical vitals at rest: BP 98/56 mmHg, pulse-88b/min, respirations rate-18b/min and body temperature 36.7 °C. Transthoracic echocardiography uncovered a lingering mass (Fig. 1, Panel. A), and a diagnosis of suspected myxoma was recorded. Other imaging modalities were non-remarkable. Given the complaints, patient was categorized under NYHA Class II prior surgery.

**Fig. 2:**
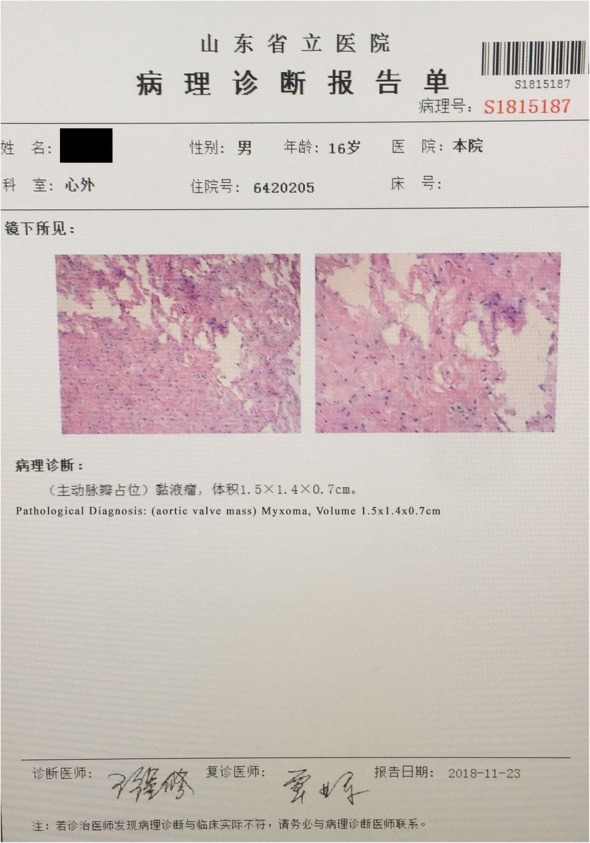
Pathological [morphologic] report of aortic myxoma

The patient was wheeled in operation room on emergency basis, and under cardiopulmonary bypass machine, after aortotomy (Panel B) a solid gelatinous mass (Panel.C) with stalk attached to right coronary cusp edge was excised and retrieved. The aortic valve and adjacent structures were intact. The aortic cavity was washed with saline to ensure complete liquidation of clots, which could potentially occlude coronary ostia. Aortotomy and chest were closed in standard fashion with two drainage tubes inserted. Prior discharge, transthoracic-echocardiography revealed no mass (Panel. D), and patient did not complain of exertional dyspnea apart from slight incisional tenderness. The morphological finding of aortic valve mass sample is reflected in Fig. 2. During his 2nd review, a month after operation, the patient had recovered and was re-categorized back to NYHA class I.

## Discussion

Cardiac myxoma (CM), a common benign primary tumor of can be categorized into syndromic (Carney Complex) and non-syndromic(isolated). Carney Complex(CNC) associated myxomas can be found in any region and system (cardiac, cutaneous, osseous, genitalia), and may manifest at a tender age [3]. While, their counter-parts, are often confined to cardiac chambers [4, 5](LA-75%, RA-18%, L/R-V-6%, valves< 1%).In addition, non-syndromic CM’s are usually asymptomatic and their discovery often incidental [6, 7], with symptoms commonly manifesting from the 5th decade of life. Aortic valve myxoma is a rare finding, and a symptomatic manifestation in a teen, as is our case is extremely unusual. In one major study of 61 cases of CM, no single aortic case was isolated. However, manifestation symptoms (dyspnea-67%, systolic murmur-49.2%) at mean ages: 48.8 and 51.9 years in males and females were observed [5, 7].

Aortic valve myxoma associated complications and potentially fatal include cardiac outflow tract obstruction [1], embolic stroke [2, 3, 8] and myocardial infarction, infective endocarditis [7] and death [3, 5]. In one study of Cardiac related deaths of 29 patients with CNC, CM accounted for 13(44.8%), and 6(20.7%) due to CM emboli [3].To the best of our knowledge, our case is 3rd case of the reported aortic valve myxoma in a teen.

## Conclusion

In view of the above, and many other reports, we advocate for immediate liquidation of the lesion, and surgical approach carry minimal risks with best outcome.

## Data Availability

Available on appropriate request from author.

## Abbreviations

CM: Cardiac Myxoma
CNC: Carney Complex
L/R: Left/right ventricle
LA: Left atrium
NYHA: New York Heart Association
RA: Right atrium
